# Supplementary material for: Embryogenic callus induction from immature zygotic embryos and genetic transformation of Larix kaempferi 3x Larix gmelinii 9
Source: PLoS One. 2021 Oct 14;16(10):e0258654. doi: 10.1371/journal.pone.0258654 (PMC8516217; doi:10.1371/journal.pone.0258654)
Supplement: S1 Table — (DOCX) [file pone.0258654.s001.docx]

| Compound | Medium composition content（mg•L^-1^） | |
| --- | --- | --- |
|  | WPM | BM |
| （一）Inorganic ion | | |
| KNO_3_ | -- | 909.9 |
| MgSO_4_•7H_2_O | 370 | 246 |
| Mg(NO_3_)_2_•6H_2_O | -- | 256.5 |
| NH_4_NO_3_ | 400 | 200 |
| K_2_SO_4_ | 990 | -- |
| MgCl_2_•6H_2_O | -- | 101.7 |
| KH_2_PO_4_ | 170 | 136.1 |
| CaCl_2_•H_2_O | 96 | -- |
| Ca(NO_3_)_2_•4H_2_O | 556 | 236.2 |
| KI | -- | 4.15 |
| MnSO_4_•H_2_O | 16.92 | 10.5 |
| ZnSO_4_•7H_2_O | 8.6 | 14.688 |
| CuSO_4_•5H_2_O | 0.25 | 0.1725 |
| H_3_BO_3_ | 6.2 | 15.5 |
| CaCL_2_•6H_2_O | -- | 0.125 |
| Na_2_MoO_4_•2H_2_O | 0.25 | 0.125 |
| FeSO_4_•7H_2_O | 27.8 | 2.78 |
| Na_2_•EDTA | 37.3 | 3.73 |
| （二）Organic matter | | |
| Nicotinic | 0.5 | 0.5 |
| Thiamine Hydrochloride | 1 | 1.0 |
| Pyridoxine Hydrochloride | 0.5 | 1.0 |
| Glycine | 2 | 2.0 |

**S1 Table. Media compound and composition content used in the text.**
