# Supplementary material for: Embryogenic callus induction from immature zygotic embryos and genetic transformation of Larix kaempferi 3x Larix gmelinii 9
Source: PLoS One. 2021 Oct 14;16(10):e0258654. doi: 10.1371/journal.pone.0258654 (PMC8516217; doi:10.1371/journal.pone.0258654)
Supplement: S1 File — (PDF) [file pone.0258654.s002.pdf]

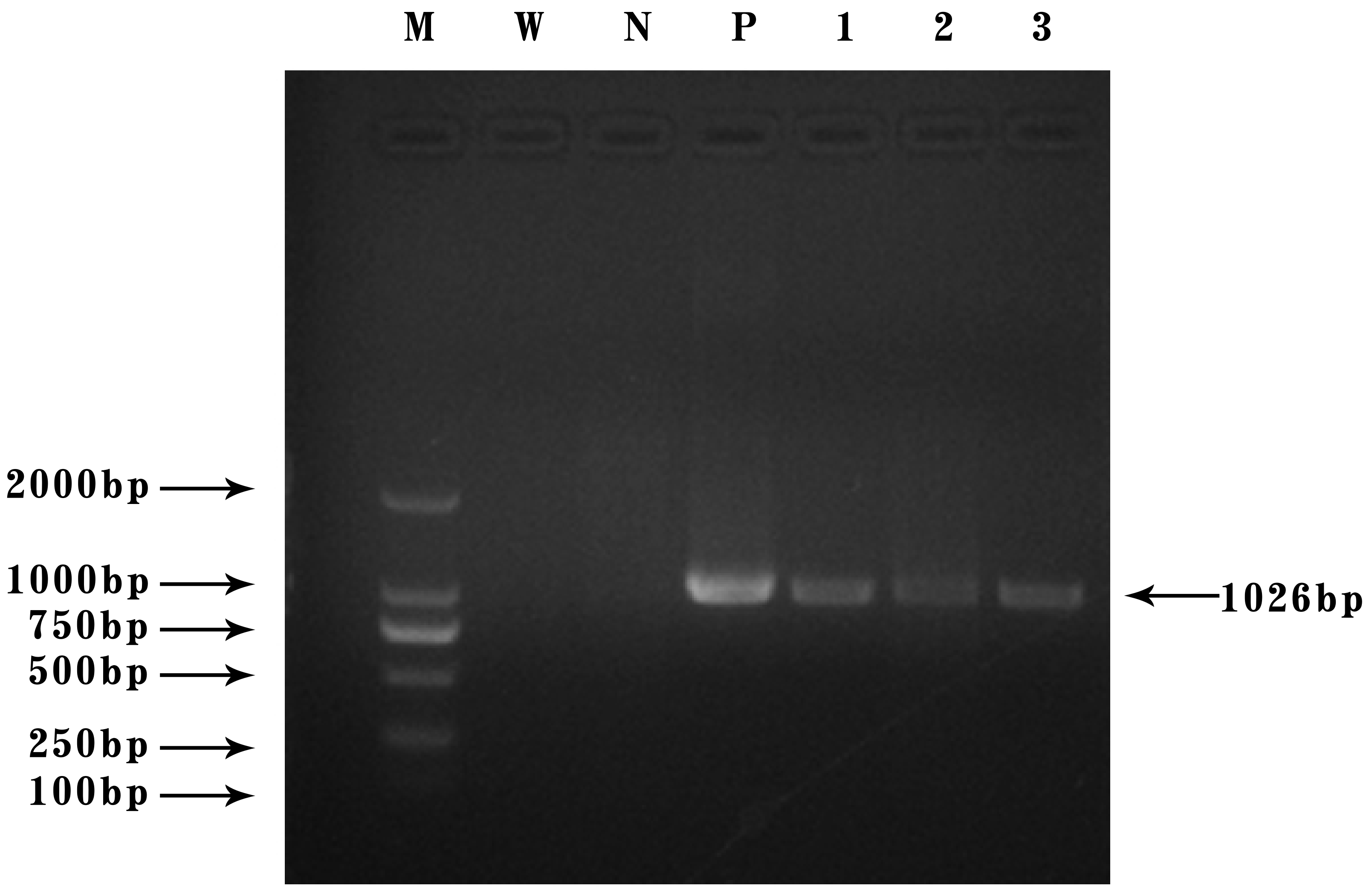

M：*GL* DNA Marker 2000(Accurate Biology, Hunan)

W ： Water

N ： Wild type callus

P ： Empty vector plasmid

1, 2, 3 ： Different transgenic empty vector callus

Fig. 10A was generated from the oiginal image
